# The male sex pheromone darcin stimulates hippocampal neurogenesis and cell proliferation in the subventricular zone in female mice

**DOI:** 10.3389/fnbeh.2015.00106

**Published:** 2015-04-29

**Authors:** Emma Hoffman, Lucy Pickavance, Thimmasettappa Thippeswamy, Robert J. Beynon, Jane L. Hurst

**Affiliations:** ^1^Mammalian Behaviour and Evolution Group, Institute of Integrative Biology, University of Liverpool, Leahurst CampusNeston, UK; ^2^School of Veterinary Science, University of LiverpoolLiverpool, UK; ^3^Centre for Proteome Research, Institute of Integrative Biology, University of LiverpoolLiverpool, UK

**Keywords:** pheromone, darcin, neurogenesis, hippocampus, subventricular zone, mice, sexual signaling, learning

## Abstract

The integration of newly generated neurons persists throughout life in the mammalian olfactory bulb and hippocampus, regions involved in olfactory and spatial learning. Social cues can be potent stimuli for increasing adult neurogenesis; for example, odors from dominant but not subordinate male mice increase neurogenesis in both brain regions of adult females. However, little is known about the role of neurogenesis in social recognition or the assessment of potential mates. Dominant male mice scent-mark territories using urine that contains a number of pheromones including darcin (MUP20), a male-specific major urinary protein that stimulates rapid learned attraction to the spatial location and individual odor signature of the scent owner. Here we investigate whether exposure to darcin stimulates neurogenesis in the female brain. Hippocampal neurons and cellular proliferation in the lateral ventricles that supply neurons to the olfactory bulbs increased in females exposed for 7 days to male urine containing at least 0.5 μg/μl darcin. Darcin was effective whether presented alone or in the context of male urine, but other information in male urine appeared to modulate the proliferative response. When exposed to urine from wild male mice, hippocampal proliferation increased only if urine was from the same individual over 7 days, suggesting that consistency of individual scent signatures is important. While 7 days exposure to male scent initiated the first stages of increased neurogenesis, this caused no immediate increase in female attraction to the scent or in the strength or robustness of spatial learning in short-term conditioned place preference tests. The reliable and consistent stimulation of neurogenesis by a pheromone important in rapid social learning suggests that this may provide an excellent model to explore the relationship between the integration of new neurons and plasticity in spatial and olfactory learning in a socially-relevant context.

## Introduction

Neurogenesis, or the generation and integration of new neurons, persists throughout life primarily in two structures of the adult mammalian brain: the olfactory bulb and the hippocampus. The precise impact of this plasticity on function in these different regions remains elusive, but neurogenesis has been implicated in a range of learning tasks including conditioned learning, memory consolidation and odor discrimination (Gould et al., 1999; Deng et al., 2010; Kageyama et al., 2012). It may also be a potential mediator of the essential balance between circuit stability and plasticity (reviewed by Lledo et al., 2006). Male odor is a highly potent stimulant of both hippocampal and olfactory bulb neurogenesis in adult female mice (Mak et al., 2007), although studies have focused on the impact of odors on neurogenesis in the olfactory system (Larsen et al., 2008; Oboti et al., 2011; Koyama et al., 2013).

New neurons added to the olfactory bulbs in adulthood are generated within the sub-ventricular zone (SVZ), the cellular layer found within the walls of the lateral ventricles in the forebrain (Luskin, 1993; Lledo and Saghatelyan, 2005). Continual production of neurons throughout life requires that this region contain a large population of neural stem cells; approximately 30,000 new cells, or neuroblasts, are produced daily within the mouse SVZ (Lois and Alvarez-Buylla, 1994). These newly generated cells must then migrate to the mature bulbar networks (Lois and Alvarez-Buylla, 1994), joining the rostral migratory stream in chains where blood vessels provide anchorage (Lois and Alvarez-Buylla, 1994; Whitman et al., 2009). Cells generated within the SVZ begin to differentiate prior to migration and continue this process as they move towards the olfactory bulb. When they reach the middle of their target, cells detach from their chains, migrate radially and begin terminal differentiation. The initial stages of proliferation and migration take approximately 7 days; cells have a mature neuronal morphology and are integrated into existing olfactory bulb circuits by day 30 (reviewed by Abrous et al., 2005), staying functional for up to a year (Winner et al., 2002).

By contrast, the germinal region for hippocampal neurons is the subgranular zone, located in the dentate gyrus deep within the hippocampus. The process of neurogenesis within the dentate gyrus comprises several developmental stages at which distinct proteins are expressed and the developing cells exhibit specific morphologies and patterns of activity (Kempermann et al., 2004). The early stages involve stem cell division followed by multiple stages of proliferative activity; over time the proliferative activity of cells decreases in conjunction with an increase in differentiation towards a neuronal fate. The final stages comprise exit from the cell cycle and the creation of dendritic connections within the existing network (Zhao et al., 2006). By 7 weeks post-generation, these cells are indistinguishable from the pre-existing neurons within the hippocampal circuitry (van Praag et al., 2002).

Exposure of adult female mice to male-soiled bedding stimulates an increase in neuron proliferation in both the hippocampus and SVZ, although soiled bedding from subordinate males is ineffective (Mak et al., 2007). Three androgen-dependent volatile pheromones in male mouse urine (2-*sec*-butyl-4, 5-dihydrothiazole and a mixture of E-E-α-farnesene and E-β-farnesene) contribute to increased cell proliferation in the female SVZ providing new neurons to the olfactory bulbs (Koyama et al., 2013); these pheromones are excreted at reduced levels by subordinate males (Harvey et al., 1989). However, while specific stimulation by odors associated with male dominance status suggests that neurogenesis may play some functional role in mate choice, the simple task of discriminating odors from dominant vs. subordinate males is unlikely to require longer-term neural plasticity. Further, any functional involvement of the hippocampus in mate assessment and selection is not yet understood, although hippocampal neurogenesis is sensitive to reproductive experience and social stress induced by intruders (reviewed by Gheusi et al., 2009).

Under naturalistic conditions, male mice deposit numerous urinary scent marks around their territories to advertise their competitive ability, important for mate selection by females (reviewed by Hurst, 2009; Roberts et al., 2014). Recently, we identified a specific pheromone in male mouse urine that underlies female attraction to spend time near male scents: a major urinary protein named darcin (MUP20; Roberts et al., 2010). This protein pheromone is involatile and must be contacted directly to stimulate attraction. It is also a potent unconditioned stimulus for rapid associative learning. After exposure in a single brief learning session, females are attracted to spend time near the airborne individual odor signature of a male associated with the pheromone (Roberts et al., 2010), an induced attraction that is remembered for at least 4 weeks (Roberts et al., 2014). Mice also rapidly learn an attraction to spend time near spatial cues previously associated with the pheromone, remembered for at least 2 weeks after a single encounter (Roberts et al., 2012). However, this learned preference for the location of darcin quickly extinguishes once females learn the pheromone is no longer present, resulting in a high degree of plasticity in learned attraction to different sites. Given the importance of male urinary scent marks in individual territory demarcation and mate assessment, and the specific role of the male darcin pheromone for regulating plasticity in olfactory and spatial learning, we tested the hypothesis that darcin is a key component in male scent for stimulating increased neurogenesis in both the hippocampus and olfactory bulbs of female mice. We also assessed whether scent needs to be consistently from the same individual male (reflecting territory ownership) to increase neurogenesis. Finally, we tested whether prior exposure to male scent over a 7 day period, sufficient to initiate increased neural proliferation, had any impact on female attraction to male scent and/or the strength and robustness of conditioned place preference for locations where females subsequently encounter male scents. As such prolonged exposure to male scent initiates a dramatic increase in neurogenesis in key brain regions involved in spatial and olfactory learning, we hypothesized that this duration of exposure might also stimulate a change in the social significance of male scent for the female even before the new neurons are incorporated into brain circuits.

## Results

### Effect of Darcin on Female Hippocampal Neurogenesis

**Figure 1:**
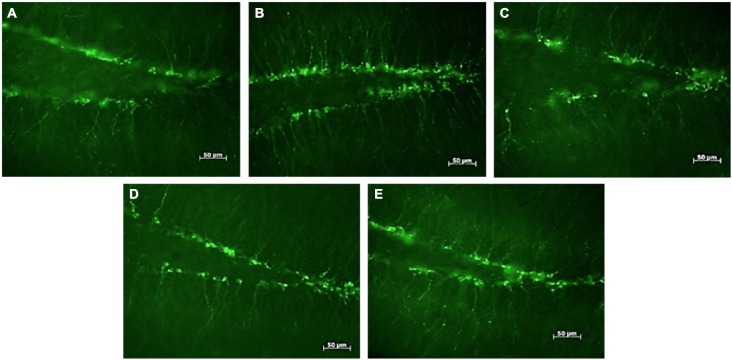
**DCX-positive cells in the subgranular zone of the hippocampal dentate gyrus of female mice**. Photomicrographs of representative responses when females were exposed to a water control **(A)**, male CD-1 urine **(B)**, male BALB/c urine **(C)**, male BALB/c urine mixed with 1 μg/μl r-darcin **(D)** or female CD-1 urine **(E)**. Mean cell counts for each treatment are shown in Figure 2A.

**Figure 2:**
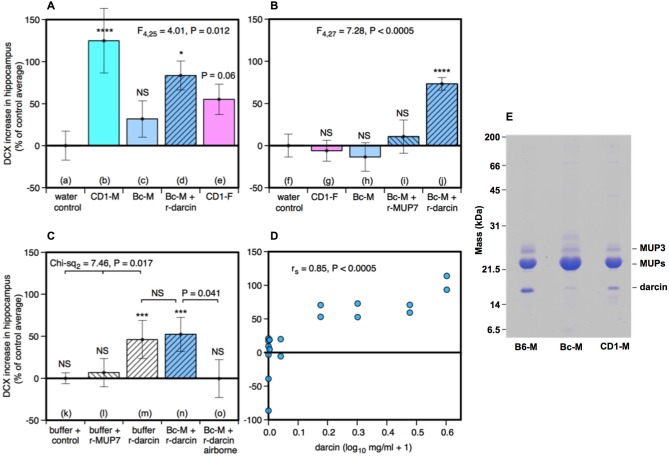
**Effect of the darcin pheromone on hippocampal neurogenesis in females**. Increase in DCX-positive cell counts in the dentate gyrus of females exposed to treatment scents for 7 days in their home cages (mean ± sem, *n* = 6 per treatment group), expressed as a percentage of the control treatment average for that experiment **(A,B)** water; **(C)** recombinant buffer; **(D)** BALB/c male urine). Females had full contact with urine stimuli (**A**: b–e, **B**: g–j, **C**: n), 1 μg/μl recombinant darcin or MUP7 control (**C**: l, m); a mixture of 1 μg/μl recombinant MUP and urine with full contact (**A**: d, **B**: i, j, **C**: n) or with access to airborne odor only (**C**: o). CD1-M: CD-1 male urine; Bc-M: BALB/c male urine; CD1-F: CD-1 female urine; B6-M: C57BL/6 male urine. ANOVA analyses established differences between treatments within each experiment **(A,B)** parametric, **(C)** non-parametric), with planned comparison of each scent treatment to the control group (*P < 0.05, **P < 0.01, ***P < 0.005, ****P < 0.001). Relationship between contact with different concentrations of darcin added to BALB/c male urine (*n* = 6 females at 0 μg/μl, *n* = 2 females at 0.01, 0.1, 0.5, 1, 2, 3 μg/μl) and DCX-positive cell count assessed using Spearman rank correlation **(D)**. SDS-PAGE illustrating differing amounts of darcin in male urine from different laboratory strains relative to the main MUPs band, with 5 μg protein loaded for each strain **(E)**. Darcin runs abnormally quickly on SDS-PAGE (Armstrong et al., 2005) while MUP3 is glycosylated and runs more slowly than other MUPs. By loading a large amount of protein, the very small amount of darcin in BALB/c male urine (<0.5%) is evident, though below threshold for response.

Seven days of exposure to bedding soiled by adult male mice in the home cage stimulates a substantial increase in neurogenesis in the hippocampal dentate gyrus of adult female mice (Mak et al., 2007). To establish (a) whether the components of male odors that stimulate this increase are located in urine, which competitive adult male mice use to scent mark their territories; and (b) whether increased neurogenesis depends on the presence of the sex pheromone darcin in male urine, we used females of the CD-1 strain in which Mak et al. (2007) had originally demonstrated the effect of prolonged exposure to male scent on female neurogenesis. However, in this study naïve female mice were exposed to urine scent only and from donors with known differences in urinary darcin content. Exposure consisted of 250 μl urine or a water control applied to clean nest material placed in female home cages over a 7 day period, refreshed on days 1, 3 and 5 (see section Materials and Methods for full details). Doublecortin (DCX), expressed almost exclusively in developing neurons, was used as a marker for neurogenesis in the subgranular zone of the hippocampus. The number of DCX-positive cells differed significantly between females exposed to different types of urine (*F*_4,25_ = 4.01, *P* = 0.012; Figures 1, 2A). Exposure to urine from singly housed CD-1 males stimulated a dramatic increase, with approximately 120% more cells expressing DCX than control females (Figure 2Ab). SDS-PAGE analysis confirmed that CD-1 strain males express normal adult male levels of darcin, though a little lower than that typical of wild or C57BL/6 strain males at approximately 8% of total urinary MUP output (Figure 2E). By contrast, BALB/c males express extremely low levels of the darcin pheromone (<0.5% of total MUP output, Roberts et al., 2010, Figure 2E), although they have normal expression of other known male-specific pheromones (Willse et al., 2006; Röck et al., 2007; Haga et al., 2010). Urine from singly housed BALB/c males failed to stimulate a significant increase in neurogenesis (Figure 2Ac). However, addition of recombinant darcin protein (r-darcin, 1 μg/μl) to BALB/c male urine stimulated an 80% increase in DCX-positive cells relative to control females exposed only to water (Figure 2Ad). This increase did not differ significantly from that stimulated by CD-1 male urine (*t*_10_ = 0.98, *P* = 0.35). Nonetheless, individual response to unmanipulated urine from CD-1 males varied more widely (range 30–293% increase above control average) than that when a standard amount of darcin was added to urine from inbred BALB/c males (range 18–140% above control average). The amount of darcin in unmanipulated urine is likely to have been more variable because of natural differences in urine dilution and/or individual differences in the amount of darcin expressed between donors. However, it is also possible that urine donors from the outbred strain differed more widely than BALB/c males in other urinary components that contributed to differences in responsiveness.

Surprisingly, there was a non-significant tendency for the number of DCX-positive cells to increase among females that were exposed to urine from other females (Figure 2Ae). This was unexpected, as bedding soiled by females of the same strain consistently failed to increase hippocampal neurogenesis in an earlier study by Mak et al. (2007). So their urine had been included as a negative control stimulus. Female mice do not normally express darcin, though trace levels can be expressed by some individuals (Cheetham et al., 2009). To check this response, we repeated this test in our next experiment using urine from other CD-1 females and found no increase relative to control females (Figure 2Bg). The slight increase in hippocampal immature neurons in response to female urine across all replicates did not differ significantly from water (*t*_22_ = −1.40, *P* = 0.09), and was clearly less than that stimulated by male urine containing darcin (*t*_22_ = −3.16, *P* = 0.005).

Response when r-darcin was added to BALB/c male urine could have been due to an overall increase in MUP concentration and/or to the presence of an unfamiliar MUP that female mice do not normally express themselves (Armstrong et al., 2005; Cheetham et al., 2009), rather than a specific response to the darcin pheromone itself. To test this, our second experiment compared response to BALB/c male urine with the addition of either r-darcin or another male-specific MUP, r-MUP7 (Mudge et al., 2008). Addition of r-darcin to BALB/c male urine again stimulated a consistent increase in DCX-positive cells relative to a water control treatment (Figure 2Bj), at a level very similar to our first set of tests (Figure 2Ad), while addition of r-MUP7 (Figure 2Bi) or BALB/c male urine alone (Figure 2Bh) was not effective. Thus, increased hippocampal neurogenesis was stimulated specifically by darcin rather than the presence of an unfamiliar recombinant MUP or increased MUP concentration.

Darcin is an involatile protein and nasal contact is required to stimulate behavioral responses to this pheromone (Roberts et al., 2010, 2012). However, darcin also binds volatile pheromones in male mouse urine and slows their release, influencing airborne volatiles that emanate from scent marks (Armstrong et al., 2005; Roberts et al., in prep). Our next experiment tested whether contact with urine containing darcin was necessary to promote neurogenesis or whether darcin acts through its influence on airborne volatiles such as 2-*sec*-butyl-4, 5-dihydrothiazole (Koyama et al., 2013). Females were given 7 days exposure to BALB/c male urine mixed with a standard amount of r-darcin (1 μg/μl) presented either inside a mesh container to prevent direct contact or held outside the container to allow contact. Females exposed only to airborne odors over 7 days failed to show any increase in neurogenesis above control females exposed to the buffer used to dissolve recombinant proteins (Figure 2Co). By contrast, those able to contact male urine containing darcin showed an increase (Figure 2Cn) that was similar in magnitude to preceding experiments (Figures 2Ad,Bj). We also tested whether darcin alone could stimulate this response without being associated with other urinary components. The protein pheromone was just as effective whether presented on its own or mixed into male urine (Figures 2Cm,n); by contrast, contact with the control r-MUP7 alone failed to stimulate any increase in neurogenesis compared to a buffer control (Figure 2Cl). Thus, the influence of darcin on hippocampal neurogenesis appears not to depend on other cues in male urine.

To establish the threshold level of darcin that was sufficient to stimulate neurogenesis, and to test whether the level of female hippocampal neurogenesis can be increased by greater male investment in this pheromone, we exposed females to BALB/c male urine containing 0, 0.01, 0.1, 0.5, 1, 2 or 3 μg/μl r-darcin. The higher concentrations of darcin in this range (1–3 μg/μl) match typical concentrations among wild-stock male house mice under caged conditions, although some wild-stock males express even higher concentrations (Hoffman et al., unpublished data). The number of DCX-positive cells in the dentate gyrus strongly correlated with the amount of r-darcin added to BALB/c male stimulus urine across all concentrations tested (*r*_s_ = 0.85, *P* < 0.0005). No increase was apparent among females exposed to only 0.01 or 0.1 μg/μl r-darcin, but strong responses were evident in all females exposed to 0.5 μg/μl or above (Figure 2D). Thus, the threshold for promoting hippocampal neurogenesis appears to lie between 0.1–0.5 μg/μl (~5–25 μM) darcin. Looking only at females exposed to levels above the threshold for response (0.5 μg/μl or more), the number of DCX-labeled cells increased with increasing r-darcin concentration (*r*_s_ = 0.68, *P* = 0.03). Thus, male urine containing relatively high levels of darcin (though well within the natural range) stimulated the strongest increase in neuronal proliferation, suggesting that the amount of male investment in this pheromone may be important.

### Darcin Stimulates Cell Proliferation in Female Subventricular Zone

**Figure 3:**
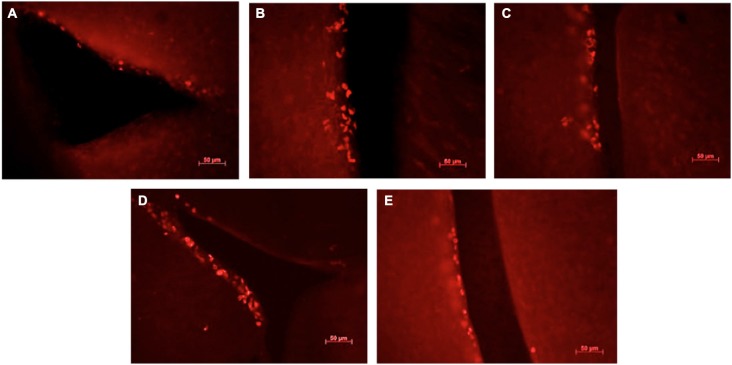
**Ki67-positive cells in the sub-ventricular zone of the lateral ventricles in female mice exposed to different scents**. Photomicrographs of representative responses when females were exposed to a water control **(A)**, male CD-1 urine **(B)**, male BALB/c urine **(C)**, male BALB/c urine mixed with 1 μg/μl r-darcin **(D)** or female CD-1 urine **(E)**. Mean cell counts for each treatment are shown in Figure 4A.

Although not the primary focus of this study, we also assessed cell proliferation in the subventricular zone (SVZ) to determine whether the same components of male urine that promote neurogenesis in the hippocampus also increase the number of new cells available to migrate into the main and accessory olfactory bulbs. We used Ki67 as a reliable endogenous marker of cell proliferation in this region (Kee et al., 2002; Figure 3).

**Figure 4:**
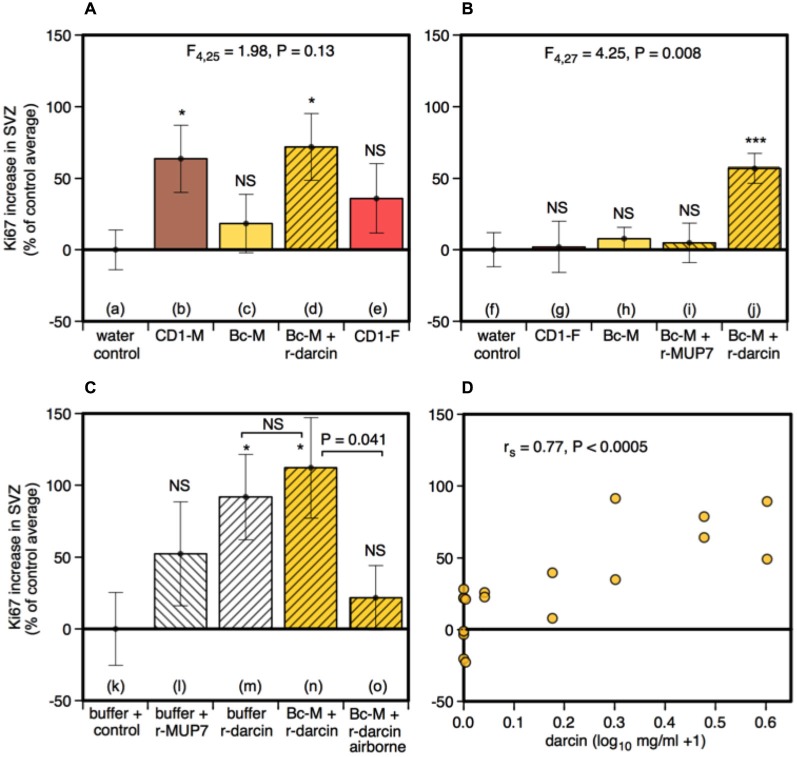
**Effect of the darcin pheromone on cell proliferation in the SVZ of female mice**. Increase in Ki67-positive cell counts in females exposed to treatment scents for 7 days in their home cages (mean ± sem, *n* = 6 per treatment group), expressed as a percentage of the control treatment average for that experiment **(A,B)** water; **(C)** recombinant buffer; **(D)** BALB/c male urine). Females had full contact with urine stimuli (**A**: b–e, **B**: g–j, **C**: n), 1 μg/μl recombinant darcin or MUP7 control (**C**: l, m); a mixture of 1 μg/μl recombinant MUP and urine with full contact (**A**: d, **B**: i, j, **C**: n) or with access to airborne odor only (**C**: o). CD1-M: CD-1 male urine; Bc-M: BALB/c male urine; CD1-F: CD-1 female urine. ANOVA analyses established differences between treatments within each experiment **(A,B)** parametric, **(C)** non-parametric), with planned comparisons of greater responses to scent treatments than to the control group (*P < 0.05, **P < 0.01, ***P < 0.005, ****P < 0.001). Relationship between contact with different concentrations of darcin added to BALB/c male urine (*n* = 6 females at 0 μg/μl, *n* = 2 females at 0.01, 0.1, 0.5, 1, 2, 3 μg/μl) and Ki67-positive cell count assessed using Spearman rank correlation **(D)**.

Male urine that contained darcin (either naturally in CD-1 male urine or artificially added as a recombinant protein to BALB/c urine) stimulated an increase in SVZ cell proliferation compared to water controls (*t*_16_ = −2.75, *P* = 0.007), while urine without darcin did not (female urine or BALB/c male urine without added r-darcin; *t*_16_ = −1.13, *P* = 0.14) (Figure 4A). Our second experiment confirmed that SVZ cell proliferation increased specifically when r-darcin was added to BALB/c male urine; addition of r-MUP7 or buffer to BALB/c male urine had no effect (Figure 4B). As with hippocampal neurogenesis, contact with the male stimulus was essential for increased SVZ cell proliferation (Figures 4Cn,o). Further, r-darcin appeared to be just as effective whether presented in the context of male urine or on its own (Figures 4Cm,n). However, cell proliferation responses measured by Ki67 in the SVZ tended to be more variable between individual females than DCX responses in the dentate gyrus. This may have reflected the different markers used in these two separate regions; Ki67 is a cellular marker for proliferation of all cell types, and does not exclusively label neurons. Because of this individual variation in response, the threshold level of darcin required to stimulate proliferation in the SVZ was less clear-cut but was approximately 0.5–1 μg/μl (25–50 μM; Figure 4D).

### Duration of Exposure, Neurogenesis and Spatial Learning

The male pheromone darcin is the key component of male mouse urine that stimulates increased proliferation of neurons in the dentate gyrus of adult females, and also increases neuronal precursors available to migrate into the olfactory bulbs. From a behavioral perspective, darcin is notable for its special ability to induce rapid associative learning of both spatial cues and airborne odors, leading to conditioned place preference for the remembered location of the pheromone (Roberts et al., 2012) and learned attraction to the individual odor signature of the pheromone owner (Roberts et al., 2010, 2014). However, there is a considerable difference in the exposure required to stimulate a behavioral or cellular proliferation response. Spatial and odor learning occurs in a single brief learning trial, involving just a few seconds of contact with darcin. By contrast, increased neurogenesis requires prolonged exposure to male scent over more than 2 days (Mak et al., 2007; Larsen et al., 2008).

**Figure 5:**
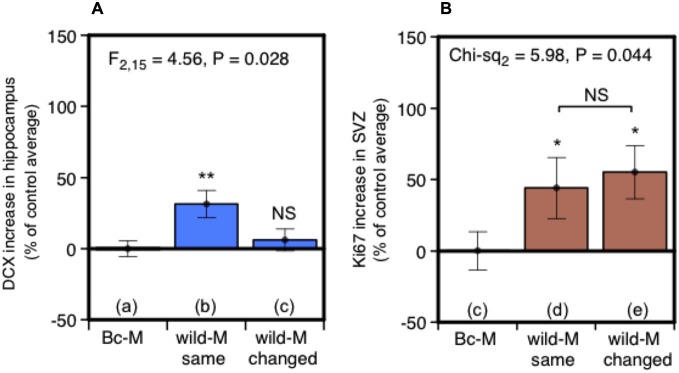
**Effect of exposure to urine from the same male or sequentially changing males on hippocampal neurogenesis and SVZ cell proliferation**. Increase in DCX-positive cell counts in the dentate gyrus **(A)** or Ki67-positive cells in the SVZ **(B)** in females exposed for 7 days to urine from the same wild-stock male (refreshed days 3, 5) or from three different wild-stock males in succession (changed days 3, 5), expressed as a percentage of the average cell count among control group females exposed to BALB/c male urine containing negligible darcin (mean ± sem, *n* = 6 per treatment). ANOVAs established differences between treatments within each experiment **(A)** parametric, **(B)** non-parametric), with planned comparisons of greater response to wild stock male urine than to the control group (*P < 0.05, **P < 0.01).

Under natural conditions, prolonged exposure may signify scents that are from the male territory in which the female resides. Such “home” scents are likely to have different social and learned significance compared to scents from other males. This led us to test whether consistent exposure to scent from the same individual male is a significant factor in stimulating neurogenesis. Two treatment groups of females were exposed to male urine in their home cages. As before, urine was replenished every 2 days over a 7-day period (days 1, 3, 5), but in this case females in one group (same male) received replenished urine that was consistently from the same individual donor while the other group (changing male) received urine from three different individuals replenished in succession. To ensure that scents were individually distinctive, urine donors were a set of three unrelated outbred wild-stock males, singly housed, that each acted as “same” and “changing” male donors for different females. Females in a control treatment group were exposed at the same time points to urine from BALB/c males without darcin. DCX-positive cells in the dentate gyrus of the hippocampus increased after exposure to urine from a single wild male donor compared to control females, but there was no increase when wild male donors changed every 2 days (Figure 5A). By contrast, cell proliferation in the SVZ increased to a similar level whether urine came from the same or changing males (Figure 5B), suggesting that the individual ownership of male scents may have a different functional impact in these two regions. Although it may appear from Figure 5A that urine from a wild-stock male stimulated less of an increase in hippocampal neurogenesis than urine from laboratory strain males (Figures 2Ab,d), this was because total DCX cell counts differed between each experiment even among control females (see section Data Analysis). This may have been due to variability between different batches of antibody used in each experiment, or differences in responsiveness between batches of females despite standardising husbandry conditions as far as possible. Thus, data were expressed relative to the average cell count among control group females for that particular experiment. In fact, the total unadjusted DCX cell count among females exposed to a single wild male donor in this experiment was slightly higher (mean 4788 ± 349 sem) than for those exposed to CD-1 male urine (3583 ± 613) or BALB/c male urine supplemented with darcin (2924 ± 275) in our first experiment shown in Figure 2A. However, as the unadjusted DCX cell count was also higher in response to a control BALB/c male urine stimulus without darcin compared to the first experiment (*t*_10_ = −3.91, *P* = 0.003), the percentage increase above control urine without darcin appears slightly weaker but cannot be compared directly.

**Figure 6:**
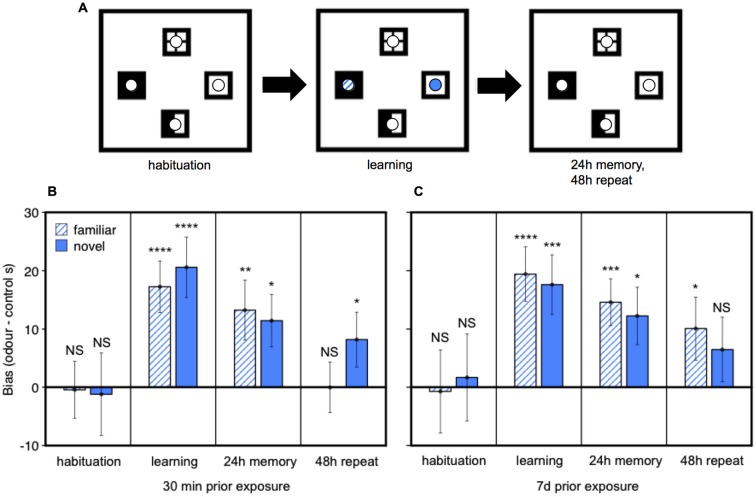
**Effect of prior exposure on female attraction to male urine and conditioned place preference responses**. After pre-exposure in the home cage to urine from a wild-stock male, females were tested in a clean arena with four locations marked by visually distinctive floor tiles bearing small petri dishes **(A)**. On the learning day, one dish contained male urine streaks familiar from the home cage treatment, one dish contained urine from a novel male and two dishes contained water streaked as a control. Conditioned place preference tests were conducted after 24 h and 48 h when all dishes contained water streaks. Data show the bias in time spent in urine locations minus the average time spent in control locations (mean ± sem, *n* = 19 per treatment) among females pre-exposed in the home cage to male urine for 30 min **(B)** or 7 days **(C)**. Asterisks indicate greater bias towards a urine stimulus location than water controls (*P < 0.05, **P < 0.01, ***P < 0.005, ****P < 0.001).

Finally, we tested whether prior home cage exposure to one male’s urine over an extended period (7 days), compared to only brief home cage urine exposure (30 min), changes female attraction to male urine and/or strengthens the place preference response conditioned by darcin in a novel arena. Seven days exposure to male urine containing darcin is sufficient to initiate the first stages of increased neural proliferation in both regions of the adult female brain, suggesting that such prolonged exposure to a male’s scent may have some specific social significance for females. Thus, this experiment addressed whether female behavioral attraction to a male’s urine also changes after 7 days of exposure, coincident with this initiation of increased neurogenesis, indicating a change in the social relevance of such scents in attracting females before the newly stimulated neurons mature and become integrated into brain circuits. Following prior exposure to urine from a wild-stock male in the female’s home cage, we tested female attraction to urine from the same male and to urine from a novel male compared to two control water locations presented in a four location conditioned place preference test (Figure 6A). During the learning session when urine cues were present, females showed equal attraction towards urine from familiar and novel males (*F*_1,36_ = 0.04, *P* = 0.84), both of which were strongly preferred to control water dishes (Figures 6B,C: learning). The duration of prior exposure to male urine in the home cage had no influence on the bias that females showed towards male urine over control dishes in this learning session (*F*_1,36_ = 0.01, *P* = 0.94; interaction between prior exposure and urine familiarity, *F*_1,36_ = 0.50, *P* = 0.48; Figures 6B,C). Female contact with darcin during the learning session thus appeared to be very similar for familiar and novel urine stimuli and was not influenced by the duration of prior exposure in the home cage. When placed back in the test arena with no urine present 24 h after the learning session, females showed a significant preference for the remembered location of male urine stimuli over control water locations as expected (*F*_2,72_ = 11.13, *P* < 0.0001). The same strength of preference was evident (Figures 6B,C: 24 h memory), regardless of the duration of prior home cage exposure (*F*_1,36_ = 0.03, *P* = 0.86) or whether urine was familiar or novel (*F*_1,36_ = 0.51, *P* = 0.48; interaction between prior exposure condition and urine familiarity, *F*_1,36_ = 0.01, *P* = 0.93). To assess the robustness of their remembered preference for male scent locations, we also compared the extinction of response when the conditioned preference test was repeated in the same clean arena the following day. Although conditioned preference can be remembered for a prolonged period, this extinguishes rapidly once females discover that there is no longer any male odor in the remembered site (Roberts et al., 2012). Although some females spent more time in the remembered location of some male scents during this second test (Figures 6B,C: 48 h repeat), an overall conditioned preference for male scent locations was no longer evident (*F*_2,36_ = 2.17, *P* = 0.12). Further, there was no evidence that preference was more persistent among females with 7 day pre-exposure to male scent compared to only 30 min for the location of either the familiar urine (*t*_36_ = 1.46, *P* = 0.15) or novel urine (*t*_36_ = −0.24, *P* = 0.81; interaction between prior exposure condition and urine familiarity, *F*_1,72_ = 1.58, *P* = 0.21).

## Discussion

The darcin pheromone produced by adult male house mice is a potent stimulus for promoting the proliferation of new neuronal cells in the female dentate gyrus. Indeed, male urine without this pheromone was ineffective, while the pheromone itself stimulated strong proliferation whether presented in male urine or on its own. This suggests that darcin is the specific pheromonal component of male mouse urine that increases neuronal proliferation in this region of the female brain. In this study, we assessed only the number of new immature neurons in the subgranular zone of the dentate gyrus and, thus, only the initial stage of neurogenesis. We do not yet know what influence darcin may have on the integration of cells into neuronal circuits. However, exposure to male scent cues in soiled bedding results not only in increased proliferation of immature neurons after a few days but also an increase in the number of mature new neurons in the dentate gyrus 4 weeks later (Mak et al., 2007). It is very likely that the proliferation of newborn neurons induced by darcin leads to an increased number of neurons as they mature. Nonetheless, it will be important to establish whether other components of male urine might also contribute to the subsequent fate of immature neurons and their eventual integration into neural circuits.

Darcin expression above 0.1 μg/μl was necessary to enhance early hippocampal neurogenesis. This is well within the normal concentration of this pheromone in the urine of wild-stock house mice (Hoffman et al., unpublished data). Above this threshold, the number of immature neurons in the dentate gyrus increased with the amount of darcin in male scent, suggesting that the level of male investment in this pheromone could be important. In some species, pheromone concentrations can influence the strength of innate responses among conspecifics (Jones and Hamilton, 1998; Sumpter and Beekman, 2003; Martín and López, 2010). As the production of a protein pheromone is metabolically costly (Gosling et al., 2000), in addition to other high costs associated with producing a pheromone that induces competitive challenges from other males (Kaur et al., 2014), the amount of darcin investment in male scent may be indicative of male quality. MUP production is under multi-hormonal control, with male-specific MUPs being androgen-dependent (Knopf et al., 1983). As testosterone is often associated with dominance, aggressiveness and the ability of males to defend their territory (Zielinski and Vandenbergh, 1993), darcin levels may also reflect subtle underlying individual differences in competitive ability.

In addition to effects in the hippocampus, darcin induced cellular proliferation in the SVZ supplying new neurons that migrate to the olfactory bulbs. Darcin, like other MUPs, is a lipocalin with a central cavity that binds hydrophobic volatile compounds in male mouse urine, slowing their release from scent marks (Hurst et al., 1998; Cavaggioni et al., 2006; Kwak et al., 2012). Darcin itself has a strong affinity for one particular volatile pheromone expressed in the urine of adult male mice: 2-*sec*-butyl-4, 5-dihydrothiazole (2HSBT; Armstrong et al., 2005; Phelan et al., 2014). In the first study to investigate male scent components that stimulate neurogenesis, Koyama et al. (2013) showed that both 2HSBT and a mixture of E-E-α-farnesene and E-β-farnesene (which are produced at high level in the preputial glands of dominant male mice) were capable of increasing cell genesis in the SVZ of adult female mice. Thus, we expected that intact urine from BALB/c male mice would be effective in stimulating SVZ cell proliferation in our study, as males of this strain express high levels of 2HSBT and farnesenes (Willse et al., 2006; Röck et al., 2007) even though their expression of darcin is negligible (Roberts et al., 2010). However, no increased proliferation was seen unless darcin was added to BALB/c male urine. This apparent mis-match in findings might be due to a key difference in the presentation of stimuli between the two studies. Koyama et al. (2013) applied a high concentration of 2HSBT, or a mixture of the two farnesenes, directly onto the nares of females twice per day over a 7 day period, repeatedly exposing females to high levels of these pheromones regardless of choice. This was important for establishing that these volatile pheromones are capable of enhancing cell proliferation in the SVZ. However, as our interest was in the natural responses of females to different male odor stimuli, we allowed females free investigation of stimuli introduced into the home cage. Thus, they could control their own exposure to the scent cues to a large extent, particularly to scents that were actively delivered to the vomeronasal organ. An extremely low level of darcin in BALB/c male urine could have reduced female exposure to volatile pheromones through two different routes. First, females are not attracted to spend much time near BALB/c urine unless darcin is added artificially (Roberts et al., 2010), thus females were unlikely to have spent long near this urine after initial investigation. Secondly, as most 2HSBT in normal male urine is bound to darcin and this substantially slows its release from drying urine (Armstrong et al., 2005), there is likely to have been rapid loss of this volatile in the absence of darcin (Hurst et al., 1998), reducing its persistence in the home cage. Addition of r-darcin to BALB/c urine is likely to have increased female exposure to this and other volatile ligands, possibly also playing a role in transporting the ligand to relevant olfactory and vomeronasal receptors. Notably, though, recombinant darcin alone was sufficient to enhance cell proliferation in the SVZ without any urinary volatiles. Thus, darcin, 2HSBT and preputial farnesenes are each capable of inducing enhanced cell proliferation in the SVZ, where new neurons are generated to migrate into the olfactory bulbs, but the presence of darcin in male scent may be key for active regulation of exposure by the female herself. Further work will be needed to understand the interaction between darcin, other male pheromones and female behavior in controlling exposure to these cues, and the consequences for subsequent integration of mature interneurons in the main or accessory olfactory bulbs.

Scent consistently from the same individual male appears to be important for stimulating the initial stages of hippocampal neurogenesis (at least when exposed to scents from genetically heterogeneous males rather than from a laboratory strain). This implies that additional information in male scent concerning individual identity modulates the response to darcin, providing additional insight into the likely functional significance of this hippocampal response. Under natural conditions, prolonged exposure to darcin in scent from the same individual male may signify a male that is successfully defending and scent-marking a local territory that the female visits regularly or, more specifically, owns the territory in which a female resides. Thus, constancy of scent ownership over time might be important for assessing individual male success in competitive territory ownership (for mate choice or for assessment of well defended nest sites), or perhaps to allow functional discrimination of resident male scents associated with “home” over those from other familiar males. A requirement to monitor and assess individual-specific male scents spread over numerous scent locations presents a much greater cognitive challenge than simple discrimination of qualitative differences between male scents (for example, those from dominant vs. subordinate males). This has the potential to require considerable plasticity in learning and memory to follow changing locations and individual status in a dynamic social system.

As prolonged (7-day) exposure to darcin in urine from a specific male initiates a major change in neuronal proliferation, suggesting a change in the social significance of a male’s scent when females experience such continued exposure, we made a first attempt to examine whether such exposure also leads to a coincident change in female attraction to that specific scent or to male urine more generally. However, we found no difference in the time that females spent close to male urine, regardless of whether they had previously been exposed to urine from the same male in their home cage or whether this was for 30 min or 7 days. We also found no evidence of a stronger or more robust conditioned place preference response to male urine after 7 days” home-cage exposure compared to only 30 min. Thus, there did not appear to be a change in the attractiveness of male urine coincident with increased initiation of neurogenesis and effects may not be evident until these new neurons are integrated into brain circuits. However, it should be noted that females used in our place preference tests were older than those used to quantify the effect of male urine stimuli on the initiation of neurogenesis (6 vs. 3 months respectively). While neurogenesis occurs constitutively throughout adulthood, the overall level shows a significant age-related decline among mice, at least when housed in single sex groups (Ben Abdallah et al., 2010). It is not yet known, though, to what extent female age (or sexual experience) influences the increase in neurogenesis stimulated by male urine. It is clear that adult females find male urine attractive from puberty throughout their reproductive lifespan. Further study of the effect of age and experience on both the behavioral and neurogenesis effects of male scents could provide useful insight into the social significance of male-induced neurogenesis in the female brain. Perhaps most importantly though, integration of the new neurons generated through contact with darcin into hippocampal and olfactory bulb circuitry is likely to take several weeks. Although beyond the scope of the current study, to investigate the role that neurons induced by repeated contact with darcin in a specific male’s scent play in female spatial and olfactory learning (and the potential role of this in mate selection), future studies need to investigate how female behavior changes over the time course of neuronal integration following such exposure to male scent.

Interestingly, although neuronal proliferation in the hippocampus was sensitive to consistency in the scent from a single male, this did not appear to be the case for the proliferative response observed in the forebrain SVZ. However, caution is needed in making any direct comparisons in response between these two regions from our study. While we were able to quantify the number of immature neurons in the hippocampus using DCX, we only assessed cellular proliferation more generally in the SVZ using Ki67 as a reliable marker. While we have shown that darcin appears to be a key component in male mouse urine for stimulating proliferation in both regions, further work will be required to understand the specific effects of this pheromone on neuronal provision and integration in the olfactory system. Nonetheless, our study has shown that the darcin pheromone in male mouse urine, which is a potent stimulus for rapid spatial and olfactory social learning, is also a reliable stimulant of neurogenesis in the hippocampus and the production of new cells available to migrate to the olfactory bulbs. Female exposure to darcin appears to provide an excellent laboratory model to explore how neurogenesis underpins plasticity in spatial and olfactory learning in a socially-relevant context, and to investigate the implications of this for female sociosexual responses.

## Materials and Methods

### Subjects and Urine Donors

All work followed national and international best practice guidelines for research using animals and was approved by the University of Liverpool Animal Welfare Committee. Administration of anesthetic via intraperitoneal injection and terminal transcardial perfusion were conducted under UK Home Office Project and Personal Licenses (Animals in Scientific Procedures Act, 1986). Mice were handled using non-aversive methods (Hurst and West, 2010) and animals were provided with enrichments in their cages throughout the study.

Subject females were 152 CD-1 laboratory mice bred in-house from parents obtained from Harlan UK (sires removed prior to birth). At 3–4 weeks of age, females were pair-housed with a same-sex sibling in 48 cm × 11.5 cm × 12 cm cages (M3, North Kent Plastics Ltd., UK). Animals used for neurogenesis experiments (*n* = 114) were aged 11 weeks (±3 d) at the start of each experiment, when pairs were moved to larger (45 cm × 28 cm × 13 cm) cages containing a clear acrylic tunnel (15 cm × 5 cm), a cardboard shelter (9 cm × 11 cm × 12 cm) and a cardboard tube (4 cm × 10 cm) placed in the same orientation and location within each cage. Subject females were naïve to any experience of adult males or direct contact with their odors before the start of an experiment. Females used in conditioned place preference tests (*n* = 38) were aged 6–7 months, housed in same-sex groups of four in 45 cm × 28 cm × 13 cm cages (MB1, North Kent Plastics Ltd., UK).

Urine donors consisted of 10 adult male and 12 adult female CD-1 mice, 25 adult male BALB/c inbred mice and 9 adult male wild-stock mice bred within the laboratory, all aged 6–12 months and sexually naive. Males were housed individually in 48 cm × 11.5 cm × 12 cm cages (M3, North Kent Plastics Ltd., UK) from weaning to ensure that they were all of similar social status (modeling territory owners) and would not be of subordinate status which is unattractive to females. Females were housed in same-sex groups of four in 45 cm × 28 cm × 13 cm cages (MB1, North Kent Plastics Ltd., UK). Wild-stock males came from a large colony of outbred wild-stock males maintained at our animal facility, where new genetic stock is frequently introduced from wild-caught animals to maintain genetic variation and wild-type behavior.

Throughout the study, subjects and urine donor animals were maintained on a reversed 12:12 light cycle (lights off at 08:00 h), housed on Corn Cob Absorb 10/14 substrate with paper wool nest material and *ad libitum* access to water and food (Lab Diet 5002 Certified Rodent Diet, Purina Mills, MO, USA). Cardboard tubes were provided to all urine donor animals for home cage enrichment.

### Stimulus Scents

Urine was collected from donors up to 5 days before the start of an experiment. Donors were confined on a mesh grid above a clean polycarbonate cage bottom for a maximum of 2 h, with cages checked for urine every 30 min. Urine was collected immediately and stored at −20°C until use. Urine from each laboratory strain was combined into same-sex, same-strain pools. Urine from genetically heterogeneous wild-stock male donors was pooled only within the same individual. When assessing female neurogenesis in response to stimulus odors, recombinant proteins were added to male BALB/c urine (1 μg/μl urine unless stated otherwise) or tested alone (1 μg/μl 50 mM phosphate, 20 mM NaCl buffer) to mimic the natural concentration of darcin observed in C57BL/6 males (approximately 10–14% of total MUP, Figure 2E) and in wild male mice (Armstrong et al., 2005; Cheetham et al., 2009).

Recombinant MUPs were expressed in *E. coli* and purified using methods described by Roberts et al. (2010). In addition to darcin (sequence located under accession numbers NP_001012323/XP_355497), we separately expressed MUP7 (accession GB/AAH91744.1) to provide another male-specific MUP unfamiliar to female CD-1 mice; this was used as a control to assess the specificity of response to the recombinant darcin pheromone. The purity of both recombinant proteins was assessed by SDS-PAGE analysis and concentration was determined by protein assay (see Roberts et al., 2010).

The amount of darcin present in male urine samples was estimated using SDS-PAGE (sodium dodecyl sulphate-polyacrylamide; Laemmli, 1970) to separate darcin from other urinary MUPs (Armstrong et al., 2005). Urine samples were diluted to 1 μg/μl protein with ddH_2_O and added in a 1:1 ratio to reducing sample buffer (100 mM DTT). Samples were centrifuged for 10 s at 9000 rpm, heated at 100°C for 5 min and centrifuged a second time. For densitometry, samples were loaded onto 15% polyacrylamide gels at a range of concentrations (0.06–1 μg/μl) to ensure that the main MUP band was not overloaded and there was a linear relationship between concentration and intensity. Electrophoresis was at a constant 200 V for approximately 45 min. Broad range molecular weight markers (Bio-Rad Laboratories Ltd., Hertfordshire) were run in a control lane. After visualising protein bands using Coomassie brilliant blue stain, the percentage of darcin present in relation to other MUPs was assessed by densitometry of scanned images using TotalLab TL100 software (TotalLab Ltd., Newcastle upon Tyne). A higher protein concentration (5 μg/μl) was loaded to provide a visual comparison between strains (Figure 2E).

### Neurogenesis Response to Scent

All neurogenesis experiments lasted 7 days and began when subjects were aged 11 weeks (±3d). Female subjects were pair housed with same-sex siblings, with *n* = 6 per treatment group. For each experiment, pairs of females were assigned at random to odor stimulus treatment groups or to an appropriate matched control group run simultaneously (ddH_2_O control group for urine stimulus treatments, a buffer control group for recombinant protein treatments). Stimulus urine, water, or buffer containing recombinant MUPs (r-MUPs), was pipetted onto clean nest material (250 μl on 7 g of nest material) and placed into the home cages of subject pairs. In an experiment where the ability to make direct contact with stimulus odor was manipulated, nesting material was presented within and around a 55 mm tea strainer (mesh holes 1 mm × 1 mm) placed into the home cage. For the contact treatment, male urine was added to the nesting material held outside the tea strainer; for airborne volatiles without direct contact, male urine was added to the nesting material inside the tea strainer. Stimulus odors were refreshed every other day (days 1, 3, 5) by replacement of old nest material with 250 μl of fresh stimulus odor on clean nest material. On the final morning (day 8) animals were removed individually from cages and anesthetized with gaseous halothane followed by a terminal intraperitoneal injection of Euthatal (65 mg/kg pentobarbital sodium solution, JM Loveridge plc, Southampton). Whilst under terminal deep anesthesia, animals were perfused transcardially with approximately 20 ml of 100 mM phosphate buffer followed by 30 ml fixative (4% paraformaldehyde, 0.2% picric acid in 100 mM phosphate buffer) using an *in vivo* perfusion system (AutoMate Scientific, Berkeley, CA). Brains were immediately dissected free and stored in fixative for 4 h at 4°C before being transferred to cryoprotectant (25% sucrose and 0.01% sodium azide in 100 mM phosphate buffer) for storage at 4°C.

### Tissue Processing

The olfactory bulbs and brain stem were dissected free from brains and 50 μm coronal sections were collected serially between coordinates Bregma +1.94 mm and −4.04 mm (Franklin and Paxinos, 2008) of the remaining tissue using a freeze microtome. This ensured all tissue in the regions of interest (lateral ventricles and hippocampus) was processed. Six sets of sections from each animal were collected free-floating in a 24-well multi-well plate. Each set contained approximately 20 complete sections that were stored in cryoprotectant (30% sucrose, 1% polyvinylpyrrolidone and 30% ethyleneglycol in 100 mM phosphate buffer) at −20°C until immunohistochemical processing. For each animal, one complete set was reserved for quantification of cells in the SVZ and one complete set was reserved for quantification of cells in the dentate gyrus.

### Immunohistochemistry

All tissue sections were washed 3 × 10 min in 10 mM phosphate buffered saline (PBS) to remove any cryoprotectant residue before being incubated overnight in the dark at 4°C with primary antibodies diluted in Triton-X solution (0.3% in 10 mM PBS) to aid penetration of the antibody into tissue. Rabbit polyclonal anti-Ki67 (1:100, Vector Laboratories, California) was used as a marker to detect proliferating cells in the SVZ in forebrain sections, and goat polyclonal anti-DCX (1:100, Santa Cruz Biotech, Texas) as a marker to detect immature neurons in the subgranular zone in hippocampal sections.

Following overnight incubation with primary antibodies at 4°C with mild agitation, tissue was washed in PBS, then incubated with secondary antibodies for 1 h at room temperature in the dark. Forebrain sections were incubated with red fluorophore-conjugated secondary antibody raised in donkey (Alexa Fluor® 594-AffiniPure (1:400), Stratech Scientific Ltd., Newmarket); hippocampal sections were incubated with green fluorophore-conjugated antibody raised in rabbit (Alexa Fluor® 488-AffiniPure (1:400), Stratech Scientific Ltd., Newmarket). After washing with PBS, 16 of the 20 sections from each of the reserved sets were mounted onto microscope slides (8 per slide) coated with a chrome-alum gelatin (CAG) solution. After air drying overnight, sections were coverslipped under anti-fade medium (Vectorshield®, Vector Laboratories, Peterborough). Thus, cell counts were performed on 16 complete sections (2 slides) for each region of interest per mouse.

Immunostaining was examined under an epi-fluorescence microscope (Zeiss AxioImager. M1) and images captured by digital microphotography (Hamamatsu ORCA I-ER digital camera, Hamamatsu photonics, Welwyn Garden City, Hertfordshire, UK) and image analysis program (Axiovision LE 4.8.2., Zeiss Imaging Systems). Immuno-positive cells were quantified in the left hemisphere of all brains. In brain tissue where total population estimates are required, counting in one hemisphere provides an accurate bilateral estimate as any sections cut with slight bias will have little impact on the overall population estimate and we confirmed in preliminary experiments that cell counts in left and right hemispheres were almost identical. Due to section thickness, the Z-stack multi-acquisition function was used to capture sets of approximately 30 images through each section. Positively stained cells were counted automatically on photomicrographs using the “Analyze Particles” tool in ImageJ version 1.4.^1^ The threshold range was set to 65–165, although in some cases the lower threshold was adjusted upwards, after manual inspection, to avoid miscounting. After excluding top and bottom images to avoid counting cells that were incomplete, Ki67- or DCX-immunopositive cells were counted on one in every ten Z-stack image to avoid repeat-sampling of the same cell in adjacent images. Subjective cell counts were also performed manually using the same photomicrographs, blind to treatment group, to verify the accuracy of automatic counts provided by ImageJ and to resolve any problems caused by clustering of labeled cells on a small number of the micrographs.

### Attraction and Conditioned Place Preference Tests

To investigate whether prior exposure to male urine over a 7-day period (sufficient to initiate neurogenesis) increases the attractiveness of male urine and/or the robustness of subsequent spatial learning induced by darcin, we compared time spent near male scents and conditioned place preference responses to male urine between females that had prior exposure to male urine in their home cages for either 7 days (*n* = 19) or 30 min (*n* = 19). In both treatment groups (females randomly assigned), each female was exposed to urine from a single individual wild-stock male donor. Genetically heterogeneous wild-stock males with different individual scent signatures were used so that we could examine responses towards familiar urine (from the same donor that females experienced in the home cage) and towards unfamiliar urine (from a different male). The same six male donors were used for both treatment groups, and to provide familiar or novel urine for different subjects, to control for any individual differences in male quality. Donors were selected on the basis that they expressed a similar quantity of darcin in their urine (approximately 1.5 mg/ml); all urine was collected from males that were housed individually and of similar age. Pre-exposure to male odor in the home cage followed the same procedure as neurogenesis experiments (250 μl male urine applied to 7 g clean nest material left in the home cage), with urine replenished every 2 days (days 1, 3, 5) for the 7-day treatment group.

Conditioned place preference tests were conducted in a white laminated MDF arena measuring 70 cm × 60 cm × 55 cm. Four acrylic tiles (14.5 cm × 14.5 cm) were fixed to the base of the arena with reusable adhesive (Blu Tack, Bostik Limited, UK), approximately 6 cm from each wall and midway along each edge. To provide internal spatial cues, the four tiles carried different visually distinctive black markings (Figure 6A). A plastic petri dish (55 mm diameter) containing 55 mm diameter glass microfibre filter paper (Whatman, grade GF/C) was sited at the center of each tile (fixed with double sided adhesive tape). The petri dishes were between 22 cm and 37 cm apart. Additional external cues were provided by the consistent location of overhead red lighting in the room and a radio receiver that provided an even background noise typical of the level heard during daily cage cleaning and animal handling procedures.

Conditioned place preference tests consisted of four daily stages, conducted 24 h apart under dim red light during the dark phase of the light cycle and recorded on DVD for subsequent transcription. The arena was cleaned thoroughly with 70% ethanol between trials, with petri dishes and filter papers discarded, but the location of all internal and external spatial cues remained consistent throughout. On day 1, 50 μl ddH_2_O was streaked on the filter paper (5 × 10 μl) within each of the four petri dishes and subjects were placed individually into the test arena for an initial 10 min habituation period to become familiar with the test arena. Day 2 consisted of a 10 min learning session with 50 μl of familiar male urine and 50 μl of unfamiliar male urine streaked in two separate clean dishes; 50 μl of control ddH_2_O was streaked in the other two dishes. The dish location of the urine stimuli was randomized between subjects but balanced as far as possible to ensure that stimuli were presented in each location a similar number of times. A 10 min conditioned place preference test was conducted on Day 3, with 50 μl ddH_2_O clean control stimuli present in all four dishes. A second 10 min conditioned place preference test, again with control water stimuli in all four locations, was repeated on Day 4 to assess extinction of response (Roberts et al., 2012). Transcription of behavior from DVD recordings was carried out blind to the position of the test stimuli during each trial, using an event recording program. We recorded total time that females spent on each tile including time spent sniffing inside the petri dishes.

### Data Analysis

All statistical tests were carried out using the IBM SPSS Statistics package (version 20).^2^ In this study, we were interested in whether exposure to specific male urine stimuli increased the amount of neurogenesis in the female hippocampus or SVZ. The baseline level of cell counts in control females that received no male scent cues differed between experiments for DCX-positive cells (*F*_2,15_ = 16.10, *P* < 0.001) but was not statistically significant for Ki67-positive cells (*F*_2,15_ = 1.51, *P* = 0.25). This could have been due to variability between different batches of antibody or to subtle differences in the experience or physiology of females between experiments despite standardised conditions. To control for differences between experiments, DCX-positive (hippocampus) and Ki67-positive (SVZ) cell counts were expressed as a percentage of the average cell count among control group females for that experiment (i.e., females randomly assigned from the same batch and tested simultaneously with a control stimulus). Thus, positive scores reflect a proportional increase in cell counts relative to females without odor exposure. We confirmed that response to the same positive stimulus (recombinant darcin in BALB/c male urine) was consistent across different experiments and batches of females both for the proportional increase in DCX-positive cells (Figures 2Ad,Bj,Cn: *F*_2,17_ = 1.07, *P* = 0.37) and for Ki67-positive cells (Figures 4Ad,Be,Cd: *F*_2,17_ = 1.56, *P* = 0.24).

Within each experiment, ANOVA analyses first tested for an overall difference between treatment groups in the proportional increase in DCX-positive or Ki67-positive cell counts. Planned comparisons examined whether cell counts in each treatment group were significantly higher than in the control group. Additional independent *t*-tests subsequently compared levels of response between specific scent treatments groups within the same experiment to assess which scent components influenced response. A Spearman’s rank test assessed whether the increase in number of immunopositive cells (as a percentage of control mean) correlated with the concentration of darcin present in urine.

In conditioned place preference tests, females were expected to spend significantly more time near a urine stimulus compared to water controls during learning sessions, and to continue to spend more time where they had previously encountered male urine if they had learned a conditioned place preference (Roberts et al., 2012). Time spent in familiar or unfamiliar urine stimulus locations, or in control locations (averaged across the two control dishes per trial), each approximated normality (assessed by Kolmogorov Smirnov and Shapiro Wilks tests, *p* > 0.05). For each test day, repeated measures ANOVA assessed whether females spent significantly longer in the location of the urine compared to the location of the control and whether there was an overall effect of scent pre-exposure on female responses.

## Author Contributions

The work was conceived by JH, LP, TT, RB who gained the funding. All authors contributed to the experimental design; EH carried out almost all aspects of practical work with advice and training from other authors and contributors. EH, JH analyzed the data. EH, JH drafted the manuscript with critical revisions from all authors. All authors approved the final manuscript for publication.

## Conflict of Interest Statement

The authors declare that the research was conducted in the absence of any commercial or financial relationships that could be construed as a potential conflict of interest.

## Footnotes

^1^http://rsb.info.nih.gov/ij/

^2^www.ibm.com
